# Identification and Functional Characterization of Anti-metastasis and Anti-angiogenic Activities of Triethylene Glycol Derivatives

**DOI:** 10.3389/fonc.2018.00552

**Published:** 2018-11-28

**Authors:** Eonju Oh, Sukant Garg, Ye Liu, Sajal Afzal, Ran Gao, Chae-Ok Yun, Sunil C. Kaul, Renu Wadhwa

**Affiliations:** ^1^Department of Bioengineering, College of Engineering, Hanyang University, Seoul, South Korea; ^2^DAILAB, DBT-AIST International Center for Translational and Environmental Research (DAICENTER), National Institute of Advanced Industrial Science & Technology, Tsukuba, Japan; ^3^School of Integrative and Global Majors, University of Tsukuba, Tsukuba, Japan

**Keywords:** Ashwagandha, TEG, TEG-derivatives, cell migration, invasion, metastasis, therapy

## Abstract

We had previously reported anticancer activity in the water extract (WEX) of Ashwagandha leaves, and identified Triethylene glycol (TEG) as an active tumor suppressor component. In this study, we investigated anti-migratory and anti-angiogenesis activities of WEX and TEG. We conducted *in vitro* and *in vivo* experiments using TEG, and its two derivatives, Triethyleneglycol dimethacrylate (TD-10), and Tetraethyleneglycol dimethacrylate (TD-11). The data revealed strong anticancer and anti-metastasis potentials in the derivatives. Non-toxic, anti-migratory doses of the derivatives showed inhibition of canonical Wnt/β-catenin axis and consequent downregulation of EMT-signaling proteins (Vimentin, MMPs and VEGF). These results endorse that the TD-10 and TD-11 have potential to safely put a check on the aggressiveness of the metastatic cells and therefore represent promising candidates for the treatment of metastatic cancers.

## Introduction

Cancer, a disease of uncontrolled cell proliferation, has become one of the biggest life-takers and is expected to account for 20 million annual new cases and related deaths by the next decade. Its therapy as well as prevention requires interventions such as chemotherapy, radiotherapy and surgery. Although the chemotherapy has been significantly successful around the world, its cost and associated undesired side effects of synthetic molecules have been raising significant concerns and contributing to the worst outcomes of the treatment. Replacing or substituting the synthetic molecules with the natural drugs has recently been considered as an option not only to reduce the burden of associated adverse effects and expenditure, but also to achieve better recovery by their holistic and harmonious effects. Out of several herbal extracts and their purified active components studied for their anticancer potentials, leaf extracts from *Withania somnifera* (Ashwagandha) have been consistently validated in several laboratories worldwide (1–6). Ashwagandha possesses a variety of withanolides, a group of secondary metabolites that consist of steroid backbone bound to lactone and/or its derivatives. Withaferin-A (Wi-A) and Withanone (Wi-N) have been consistently identified as active anticancer withanolides (1, 6–11). Wi-A is the most extensively studied and reported to cause (i) activation of tumor suppressor proteins (1, 12), (ii) inactivation of transcription factor NFκB that regulates cytokine and inflammatory response production (13), (iii) collapse of intermediate filament protein-vimentin, an important regulator of cell shape and migration (14), (iv) oxidative stress and apoptosis (6, 9, 15) and (v) inhibition of epithelial-mesenchymal transition (EMT) signaling (16).

We had earlier investigated anticancer activity in the water extract of Ashwagandha leaves (WEX) and found that it possesses considerable potentials that were validated in *in vitro* and *in vivo* models (2, 4, 17), and hence proposed beneficial for cancer treatment. Water extracts of leaves are eco- as well as bio-friendly due to the fact that the plants are not sacrificed and the organic solvents are replaced with easy, economic, convenient and safe alternative (water). Chemical analyses of WEX showed the presence of very low levels of Wi-N or Wi-A. By activity-based fractionation and NMR analysis, we identified triethylene glycol (TEG) as a key component that caused activation of tumor suppressor proteins p53 and pRB resulting in growth arrest of cells *in vitro* and *in vivo* (17).

TEG (C_6_H_14_O_4_) is a member of dihydroxy alcohol family, known to have high ability to hold water molecules (18). It has been popularly used as a disinfectant anti-bacterial, anti-fungal and desiccant in paint, oil and gas industry, and in dentistry (19). It is commercially produced by high temperature oxidation of ethylene in the presence of silver oxide, followed by hydration of ethylene oxide. We used commercially available TEG in *in vitro* assays and found it to be cytotoxic to human cancer cells (17). Molecular analyses revealed that TEG caused induction of p53 and pRB pathways resulting in G_1_/S growth arrest and apoptosis. It also caused downregulation of matrix metalloproteases (MMPs), critical mediators of cell migration and cancer metastasis, suggesting its anti-metastasis activity. An old study on dogs with metastatic tonsillar epithelioma and reticulum cell sarcoma reported the anti-tumor effect of TEG without causing any significant toxicity to animals (20). Liu et al. reported that the triethylene tetramine (TETA) as a novel ligand for G-quadruplex inhibited telomerase and induce senescence in tumor cells (21). Furthermore, it was found that although low dose of TETA caused only a weak inhibition of tumor cell growth in culture, it could significantly enhance anti-tumor activity of traditional anti-tumor drugs *in vitro* and *in vivo*. In all, TEG and some of its derivatives have previously been suggested to possess anticancer potential, the molecular mechanisms remain unknown. In the present study, we recruited 10 TEG derivatives to examine their therapeutic potential in human carcinomas.

Carcinogenesis involves inactivation of innate tumor suppressor and activation of oncogenic pathways resulting in hyper-proliferation of cells that compete with normal neighboring cells for nutrients and space (22). Various signaling pathways are involved to maintain and modulate intracellular homeostasis that eventually lead to cell cycle progression, EMT, growth arrest and apoptosis, post-crisis survival, differentiation, etc. Based upon the mechanisms such as intracellular stress, protection against the external shear, favor in secondary niche and other factors, cancer cells settle their fate. These mechanisms, therefore, are considered as attractive targets to eliminate cancer cells. Often, as the disease progresses, some cells gain metastatic properties such as loss of cell-to-cell adhesion and gain of migratory, invasive and angiogenic characteristics by activation of EMT signaling. EMT is characterized by an upregulation of migratory proteins such as MMPs, vimentin, fibronectin, and downregulation of cell adhesion proteins such as cadherins. It is largely driven by activation of Wnt/β-catenin, hypoxia, Hedgehog, Notch and TGF-β signaling. Wnt (secreted glycoprotein) binds to cell surface receptor Frizzled and regulates cytoplasmic destruction and nuclear translocation of β-catenin that controls cell proliferation and fate decisions by transcriptional activation of several proteins (23) including MMPs (24), vimentin (25), and vascular endothelial growth factor (VEGF) (26). In view of our earlier findings that the water extract of Ashwagandha leaves possesses anticancer activities and activates pRB tumor suppressor pathway, we, in the present study, investigated the effect of TEG and its derivatives on cancer cell survival and migration *in vitro* and *in vivo*. Out of the 10 derivatives analyzed, we found Triethylene glycol dimethacrylate (TD-10) and Tetraethylene glycol dimethacrylate (TD-11), could be potential anticancer drugs due to their anti-migratory activities.

## Materials and methods

### Cell culture

A549 (non-small cell lung cancer), U343 (human glioblastoma), HUVEC (human umbilical vein endothelial cells), and TIG3 (normal skin fibroblast) cells were maintained in Dulbecco's Modified Eagle's Medium (DMEM) (Invitrogen) supplemented with 10% fetal bovine serum and 1% penicillin/streptomycin in a humidified incubator (37°C and 5% CO_2_). Cells (60–80% confluency) were treated with different concentrations of Water-Extract of Ashwagandha leaves (WEX) (0.25% to 2.5%), Triethylene glycol (TEG) (0.025% = 1.65 mM to 2.5% = 165 mM) or its derivatives Triethylene glycol dimethacrylate (TD-10) (0.005% = 0.19 mM) and Tetraethylene glycol dimethacrylate (TD-11) (0.005% = 0.16 mM) as shown in Supplementary Figure S1. Cytotoxic response and molecular assays were performed typically after 48 h treatment, as described below.

### Cell viability and growth assay

Cytotoxicity of TEG derivatives was determined by MTT {3-(4,5- dimethylthiazol-2-yl)-2,5-diphenyltetrazolium bromide (Life Technologies)}, short-term-QCV, ATP-Glo™, and AlamarBlue™ cell viability assays.

#### MTT assay

One thousand cells per well were plated in 96-well plate and allowed to settle overnight, followed by treatment with varying doses of the drugs. The control or drug treated cells were incubated at 37°C and 5% CO_2_. After 48 h, 10 μL of MTT (Sigma-Aldrich, M2003-1G) in phosphate buffered saline (PBS; 2 mg/mL) was added to each well and incubated at same conditions for 4 h. The media and MTT from the wells were aspirated out and replaced with 100% DMSO, followed by measurement of optical density at 570 nm using Tecan infinite M200® Pro using a microplate reader (Infinite 200 PRO; Tecan Group Ltd., Mannedorf, Switzerland).

#### Short-term QCV assay

Two hundred thousand cells per well were plated in 12-well plate and allowed to settle overnight, followed by treatment with varying doses of the drugs. The control or drug treated cells were incubated at 37°C and 5% CO_2_. After 48 h, cells were fixed, stained and de-stained into the solution, which was quantified by the help of a photospectrometer as described previously (27).

#### AlamarBluet™ assay

One thousand cells per well were plated into 96-well plate and allowed to settle overnight, followed by treatment with varying doses of the drugs. The control or drug treated cells were incubated at 37°C and 5% CO_2_ for 48 h. 10 μL of 10X AlamarBlue™ (Thermo Scientific, DAL1100) was then added to each well and incubated for another 4 h, followed by measurement of optical density at 570 nm using Tecan infinite M200® Pro using a microplate reader (Infinite 200 PRO; Tecan Group Ltd., Mannedorf, Switzerland).

#### ATP-Glo™ assay

One thousand cells per well were plated in 96-well plate and allowed to settle overnight, followed by treatment with varying doses of the drugs. The control or drug treated cells were incubated at 37°C and 5% CO_2_ for 48 h. ATP-Glo™ detection cocktail was then added and cytotoxicity was evaluated following the manufacturer's instructions (Biotium, 30020-1).

Cell viability in each of the above experiment was calculated in percentage against the DMSO-treated control, and plotted using standard office software. SD and statistical significance were calculated by GraphPad software (2017) from three independent experiments and shown as ^*^p < 0.05, ^**^p < 0.01, ^***^p < 0.001.

In order to estimate the growth rate of cells, one million cells (each of TIG3 and A549) were plated in 6-well plates and allowed to settle overnight, followed by culture either in control or drug-supplemented medium. Cells were trypsinized from each well from each treatment group and counted at 24, 48, 72, and 96 h. Proliferation graph was plotted considering control at 0 h as 100%. Statistical significance was calculated by GraphPad software (2017) from three independent experiments and shown as ^*^p < 0.05, ^**^p < 0.01, ^***^p < 0.001, and ns = not significant.

### Colony forming assay

For long-term cell viability, colony forming assays were performed. One thousand cells were plated in a 6-well cell culture dish and cultured either in control or drug-supplemented medium. Cells were cultured for 8–10 days (until colonies appeared in control cultures) with regular change in medium on every alternate day. Colonies were fixed in methanol: acetone (1:1) and stained with 0.5% crystal violet for overnight followed by washing with milliQ to remove the excess stain. The plates were air-dried and photographed. Colonies from three wells and three experiments were counted and plotted. Statistical significance was calculated by GraphPad software (2017) from three independent experiments and shown as ^*^p < 0.05, ^**^p < 0.01, ^***^p < 0.001.

### Crystal violet staining

One hundred thousand cells were plated in a 6-well plate, and cultured either in control or drug-supplemented medium for 48 h. The cells were then fixed in 100% methanol and stained with 0.5% crystal violet for 2 h. The plates were washed and dried, followed by visualization under the microscope. Stained cells were de-stained in 15% methanol-15% glacial acetic acid in Milli-Q water solution and was quantified by the help of a spectrophotometer. Statistical significance was calculated by GraphPad software (2018) from three independent experiments and shown as ^*^p < 0.05, ^**^p < 0.01, ^***^p < 0.001.

### Flow cytometry

A549 cells were synchronized by serum starvation. Cells were cultured in 10% FBS-supplemented DMEM for 24 h, followed by 5, 1, 0, and 10% FBS-supplemented DMEM for 24 h each, successively, followed by culture in control or drug-supplemented medium for 48 h. Two hundred thousand cells per milliliter were washed twice and re-suspended in 300 μL PBS and fixed by slowly adding 700 μL ice cold 100% ethanol with mild vortex. Fixed cell samples were stored in −20°C overnight, then spun down and washed twice with PBS. RNAse (Thermo Scientific, EN0531) was added to a final concentration of 100 μg/ml and incubated at 37°C for 90 min. Suspensions were then spun down and re-suspended in 200 μl Guava Cell Cycle Reagent and incubated at room temperature for 30 min in dark, diluted in 1–2 mL PBS and taken up for data acquisition cell cycle analysis (Guava™ PCA Systems).

### Cell migration and invasion assays

Cell migration of control and drug treated cells was examined by Wound-Scratch assay. One hundred and fifty thousand A549 cells per well were plated in a 6-well plate, allowed to settle overnight. A small scratch was made using a sterile pipette tip to simulate an area devoid of cells. The cells were either cultured in control or drug-supplemented medium. Movement of cells to the scratch area was monitored under the microscope every 12 h. Photographs of scratch and migrating cells were captured every 24 h. Scratched area was calculated using ImageJ software (NIH) and plotted in percentage.

The effect of drugs on cellular chemotactic motility responding to VEGF165 was assessed using Transwell migration chambers (Corning Costar, Cambridge, MA) with 6.5-mm diameter polycarbonate filters (8 μm pore size). U343 cells were stimulated with 10 ng/mL VEGF165 and treated with Avastin or Ashwagandha reagents. After 48 h incubation at 37°C, conditioned medium was placed in the lower chamber of Transwell. HUVECs, incubated for 6 h in serum-free EGM-2, were harvested by trypsinization and loaded into the upper wells at 1 × 10^5^ cells/well. The Transwell chambers were then incubated at 37°C for 48 h following which the cells remaining on upper part of the filter were removed with a cotton swab. The filters were stained with hematoxylin and eosin (H & E). Cells that migrated through the bottom of filter membrane were identified and counted at a magnification of × 100.

Invasion assays were carried out using BD BioCoat^TM^ Growth Factor Reduced MATRIGEL^TM^ Invasion Chamber (BD Biosciences, San Jose, CA) following the manufacturer's instructions. The experiment was performed as described for the cell migration assay. After 72 h, non-invading cells were removed, and the invading cell population under the surface of filter was fixed and stained. The membranes were mounted on glass slides, and invaded cells were counted at × 100 magnification.

The formation of capillary-like structures on a basement membrane matrix of HUVECs was used to assess the anti-angiogenic activity of drugs. Tissues culture wells (16-mm diameter) were coated with 250 μL of growth factor-reduced Matrigel (BD Biosciences) for 30 min at 37°C. HUVECs, serum-starved in serum-free EGM-2 for 6 h, were harvested and suspended in conditioned media that contained 50 ng/mL VEGF165. Subsequently, the suspended HUVECs were treated with Avastin or test reagents at a density of 1 × 10^5^ cells/well. HUVECs were then seeded onto the layer of Matrigel, and the cells were allowed to form vesicular tubes for 24–72 h at 37°C. Tube formation was photographed at a magnification of × 50. The area covered by the tube network was quantified by Image-Pro Plus software (Media Cybernetics, Silver Spring, MD).

### Immunofluorescence

Cells were plated on glass coverslips placed in 12-well cell culture plates. After 48 h of treatment, cells along with control were fixed in methanol: acetone (1:1). Cells were permeabilized with Tween-20 in phosphate buffered saline (PBST), washed with phosphate buffered saline (PBS), and blocked with 2% bovine serum albumin protein dissolved in PBST. Fixed cells were incubated with primary antibodies against β-catenin (Santa Cruz, sc-7963), BCL2 (Santa Cruz, sc-492), Caspase 9 (Santa Cruz, sc-7885), E2F-1 (Santa Cruz, sc-251), MMP2 (Santa Cruz, sc-10736), Mortalin (28), p53 (Santa Cruz, sc-6243), pSMAD 2/3 (Cell Signaling Technologies, 8828S), RB (Cell Signaling Technologies, 9309S), SMAD 2/3 (Santa Cruz, sc-133098), Vimentin (Santa Cruz, sc-5565), VEGF (Santa Cruz, sc-507), Cyclin D1 (Santa Cruz, sc-20044), and CDK4 (Santa Cruz, sc-260) proteins overnight, washed with PBS-PBST-PBS (5 min each), incubated with either Alexa-Fluor 488 goat anti-mouse IgG (Life Technologies, A11029) or Alexa-Fluor 594 goat anti-rabbit IgG (Life Technologies, A11037), depending upon the source of the primary antibodies, for 2 h, washed with PBS-PBST-PBS (5 min each), incubated with Hoechst 33342 stain (Invitrogen Molecular Probes, H3570) for 10 min, washed with PBST-PBS-ultrapure water (5 min each), and mounted on glass slides. The cells were then visualized for immunofluorescence under a microscope at × 400 magnification. Protein expression was quantified using ImageJ software (NIH) and plotted in percentage.

### Immunoblotting

Cell lysates from control and treated cells were prepared in RIPA buffer (Thermo Scientific, Rockford, IL) containing complete protease inhibitor cocktail (Roche Applied Science, Mannheim, Germany). Cell lysates (containing 20 μg protein) were separated on a SDS-polyacrylamide gel using Mini-PROTEAN® Tetra cell equipment (Bio-Rad, Hercules, CA), and transferred onto PVDF membrane (Millipore, Bedford, MA) by Mini Trans-Blot® Electrophoretic Transfer Cell (Bio-Rad). Membranes were blocked in 0.2% Tween-20 in TBS (TTBS) containing 3% BSA and incubated with antibodies (1–2 h), washing buffer (TTBS) (10 min × 3) and then horseradish peroxidase-conjugated secondary antibody [goat anti-mouse HRP (Santa Cruz, sc-2005) or goat-anti-rabbit HRP (Santa Cruz, sc-2004)] (45 min), washing buffer (10 min × 3) The blots were then developed using chemiluminescence (GE Healthcare, UK) and visualized using Lumino Image Analyzer equipped with CCD camera (LAS3000-mini; Fuji Film, Tokyo, Japan). Protein expression was quantified using ImageJ software (NIH) and plotted in percentage.

### Mortalin ELISA

Cell lysates, as prepared above, were plated into clear flat bottom polystyrene Costar™ 96-well plate (Corning, 3632) pre-coated with 100 ng/mL anti-mortalin antibody diluted in coating buffer (BioLegend, 421701) and diluent buffer (Biolegend, 421203), and incubated at room temperature overnight. Wells were then washed with washing buffer (0.05% Tween 20 in PBS) (1 min × 3), and reloaded with 100 ng/mL anti-mortalin antibody diluted in diluent buffer. The plate was incubated at room temperature overnight, followed by washing (1 min × 3) and incubated with 100 ng/mL horseradish peroxidase-conjugated secondary antibody goat anti-mouse HRP (Thermo Scientific, 31430) diluted in diluent buffer at room temperature for 3 h. The plate was washed (1 min × 3) and then incubated with TMB substrate (BioLegend, 421101) for 30 min for color development. Stop solution (BioLegend, 423001) was added onto the substrate and mixed. The optical density was measured at 450 nm using Tecan infinite M200® Pro microplate reader (Tecan Group Ltd., Mannedorf, Switzerland). Recombinant mortalin protein diluted in diluent buffer was used as a standard. Mortalin protein concentration (in %) was calculated against the control using the slope equation obtained in the standard.

### Anti-tumor effect

To assess the anti-tumor effect of drugs, subcutaneous U343 tumor xenografts were established by injecting two and a half million U343 cells subcutaneously into the abdominal dermis of 6- to 8-weeks old male athymic nude mice (*n* = 7). Each treatment (PBS, TD-10, or TD-11, 5%) was administered (200 μl) every alternate day for a total of 25 days intraperitoneally, until the average tumor volume reached 80 mm^3^. First day of the treatment was designated as Day 1. The length (L) and width (W) of each tumor were measured every alternate day with a caliper, and tumor volume was calculated according to the formula: tumor volume = 0.523 LW^2^. Body weight of mice was measured every alternate day. All animal experiments were performed following the protocols for animal experiments recognized and approved by the Animal Care and Use Committee, Institute of Laboratory Animal Science of Peking Union Medical College (ILAS-PG-2014-018) and by the Hanyang University Institutional Animal Care and Use Committee (2015-0002A).

### Histology and immunohistochemistry

On 26th day of the treatment tumor tissues were collected from mice. Tumor tissues were fixed in 10% formalin, processed for paraffin embedding, and cut into 5 μm sections. Each section was stained with H & E stain visualized under a light microscope and photographed. Immunohistochemistry of tumor tissue, for the assessment of VEGF or CD31, was performed by incubating the sections with mouse anti-human VEGF antibody (BD Biosciences) or rabbit anti-mouse CD31 antibody (Abcam, Cambridge, UK) at 4°C overnight. Sections were then incubated with horseradish peroxidase (HRP)-conjugated goat anti-mouse IgG antibody (Southern Biotech, Birmingham, AL) or biotinylated polyclonal anti-rabbit antibody (Vector Laboratories, Burligame, CA), followed by streptavidin-HRP antibody (BD Biosciences). Sections were then developed with diaminobenzidene (DAB; Dako, Carpinteria, CA) and counterstained with Meyer's hematoxylin.

### Hemolytic assay

A qualitative hemolytic assay to test the erythrocyte lysis property of TEG derivatives was performed. Blood extracted from a healthy mice heart was diluted with diluent buffer (0.85% NaCl containing 10 mM CaCl_2_) to obtain 2% RBC suspension. One hundred and fifty microliter suspension was mixed with 75 μL diluent buffer and 75 μL of TEG derivatives (1%) by gentle pipetting. The mixture was incubated at room temperature for 1 h, followed by centrifugation (16,000 rpm) at room temperature for 5 min. Two hundred microliter of the reaction mixtures was loaded into a 96-well plate to read the absorbance at 540 nm. Ratio of absorbance corresponded to the degree of hemolysis caused by the TEG derivatives. Triton X-100 (0.2%) in diluent buffer was taken as the positive control.

## Results

**Figure 1:**
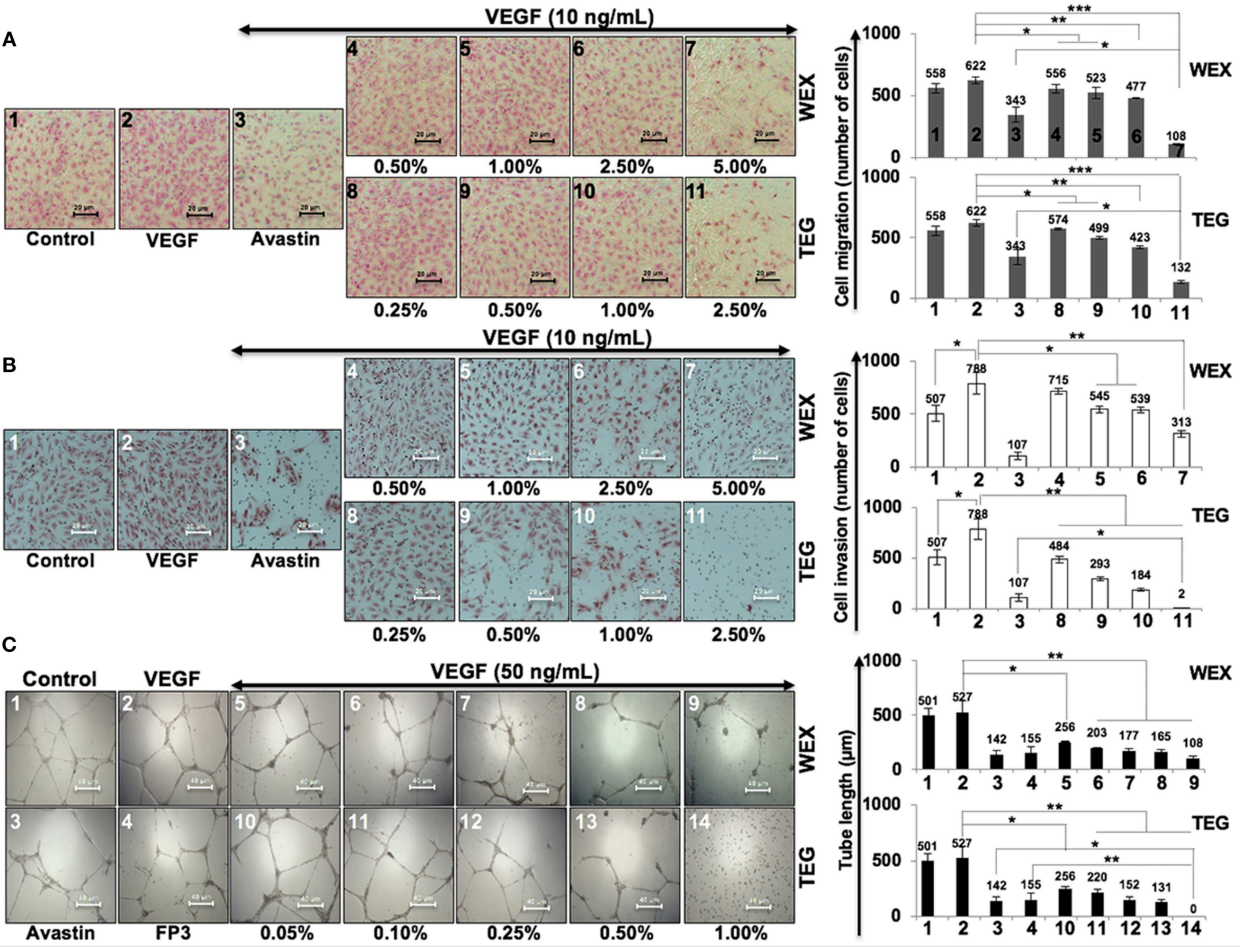
Effect of water extract (WEX) of Ashwagandha leaves and TEG on cell migration and invasion capacity of HUVECs. (**A)** VEGF-treated cells showed mild increase in migration; VEGF + Avastin (positive control)-treated cells showed inhibition. Both WEX and TEG showed dose-dependent inhibition of migration. (**B)** VEGF-treated cells showed enhanced invasion; VEGF + Avastin (positive control)-treated cells showed inhibition. Both WEX and TEG showed dose-dependent inhibition of invasion. Migrated and invaded cells were counted in three randomly selected fields. Data are shown as mean ± standard deviation (*SD*) of three independent fields/well. Maximum inhibition of cell migration was observed in cells treated with 5% WEX and 2.5% TEG. WEX (5%) and TEG (0.5 to 2.5%) caused strong inhibition of cell invasion. (**C)** Effect of WEX and TEG on tube-formation in HUVEC. VEGF-treated cells showed mild increase in tube formation capacity of HUVEC. Avastin and FP3 (positive controls), WEX and TEG showed dose-dependent inhibition of tube formation. Statistical analysis from three independent experiments is depicted as *p < 0.05, **p < 0.01, ***p < 0.001.

We had earlier identified triethylene glycol (TEG) as a cytotoxic component in the water extract of Ashwagandha leaves (17). It caused activation of p53 and pRB tumor suppressor pathways; the latter was activated preferentially in cancer cells. In the present study, we have determined the effect of TEG on cell migration and invasion capacity, the two phenotypes that are critically important for cancer metastasis. As shown in Figure 1A, we found that whereas VEGF caused mild increase in migration capacity in HUVEC, treatment with WEX (2.5 to 5.0%) and TEG (1.0%−66 mM to 2.5%−165 mM v/v) caused strong inhibition. A dose-dependent inhibition of migration was observed in which 5.0% WEX and 2.5% TEG caused inhibition that was stronger than the positive control, Avastin (10 μg/mL), a well-established clinical cancer drug with anti-metastatic and anti-angiogenic properties. We next investigated the effect of WEX and TEG on invasion capacity of HUVEC. As shown in Figure 1B, VEGF caused considerable increase in invasion capacity of HUVEC that was antagonized by Avastin. Of note, both WEX (1.0–5.0%) and TEG (0.5–2.5%) caused significant loss of VEGF-induced invasion of cells *in vitro* (Figure 1B). Both anti-migration and anti-invasion effects of WEX and TEG (1.0–2.0%) was comparable to Avastin (10 μg/mL). The results were strongly endorsed by tube formation assays in HUVEC cells. Although VEGF caused only a mild induction of tube formation, both WEX and TEG treatment inhibited the tube formation at concentrations ranging from 0.05 to 1.0%. Of note, the effect was comparable with Avastin and FP3 (VEGF blockers) that were used as positive controls (Figure 1C).

**Figure 2:**
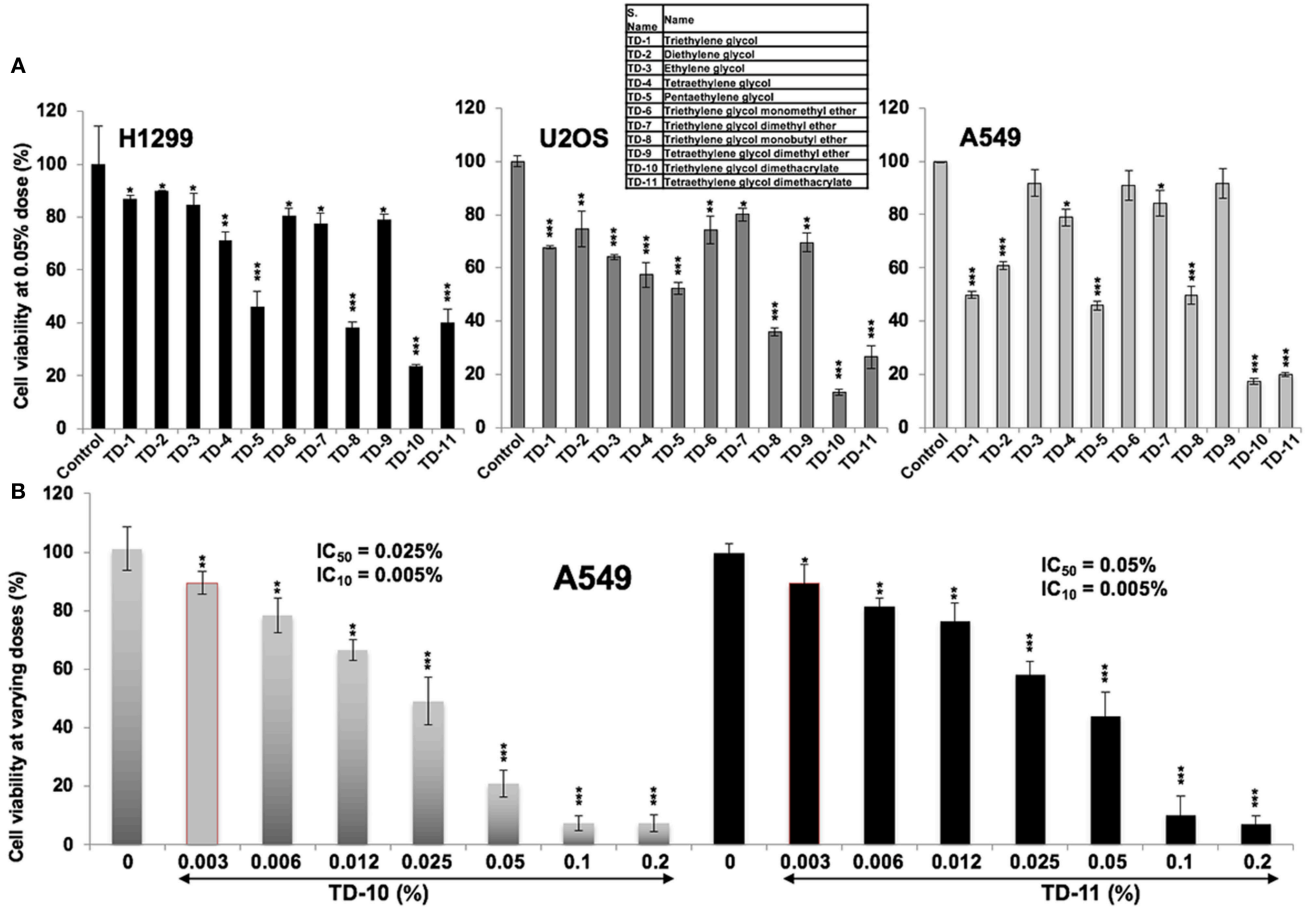
Cytotoxicity curves. (**A)** Cytotoxicity of TEG (TD-1) and its 10 derivatives (TD-2 to TD-11) (Supplementary Figure S1) on H1299, U2OS, and A549 cells; TD-10 and TD-11 demonstrated the most remarkable cytotoxicity in each cell line. (**B)** Dose dependent cytotoxicity of TD-10 and TD-11 in A549 cells is shown. Statistical analysis is depicted as *p < 0.05, **p < 0.01, ***p < 0.001.

In light of the above data, we set out to investigate the anticancer and anti-metastasis activities of 10 TEG derivatives (Supplementary Figure S1). Cytotoxicity assays revealed that pentaethylene glycol (TD-5), triethylene glycol monobutyl ether (TD-8), triethylene glycol dimethacrylate (TD-10) and tetraethylene glycol dimethacrylate (TD-11) were more potent than TEG (TD-1) in all the three cancer cell lines (Figure 2A) examined. We selected TD-10 and TD-11 for further analyses. Dose dependent cytotoxicity was observed both for TD-10 and TD-11; with IC_50_ of 0.025% = 0.95 mM and 0.050% = 1.6 mM, respectively in four kinds of cell viability assays with independent principles (Figure 2B, Supplementary Figure S2).

**Figure 3:**
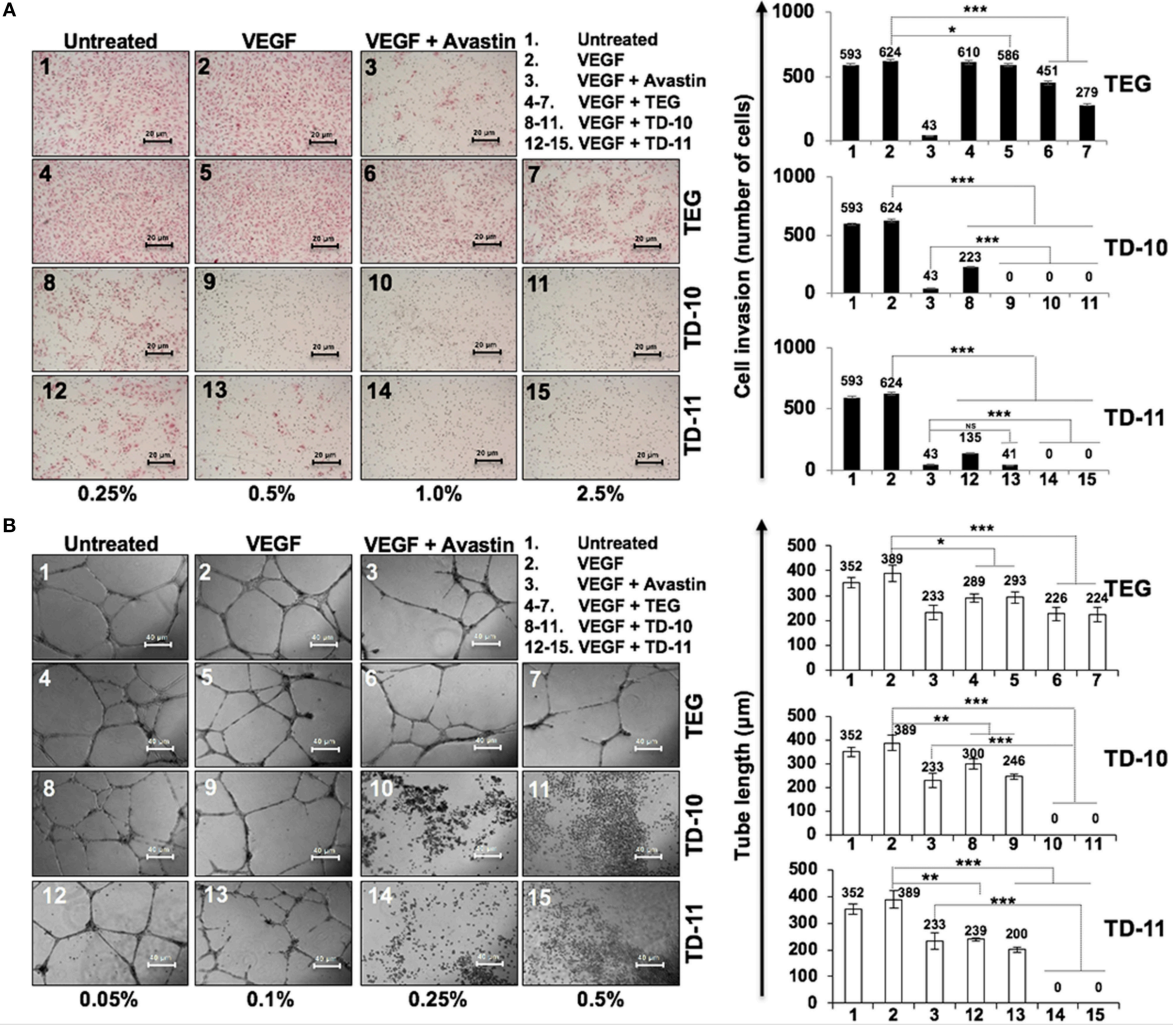
Effect of TEG and its derivatives TD-10 and TD-11 on cell invasion capacity and tube-forming capacity of HUVEC. (**A)** VEGF-treated cells showed mild increase in invasion. TEG, TD-10, and TD-11 treated cells showed inhibition of VEGF-induced invasion; TD-10 and TD-11 showed stronger inhibition than TEG. Avastin was used as a positive control. (**B)** VEGF was used to induce tube formation. TEG, TD-10 and TD-11 treated cells showed inhibition of tube formation; TD-10 and TD-11 showed stronger inhibition than TEG. Avastin was used as a positive control. Statistical analysis is depicted as significant *p < 0.05, **p < 0.01, ***p < 0.001 and NS for insignificant change.

**Figure 4:**
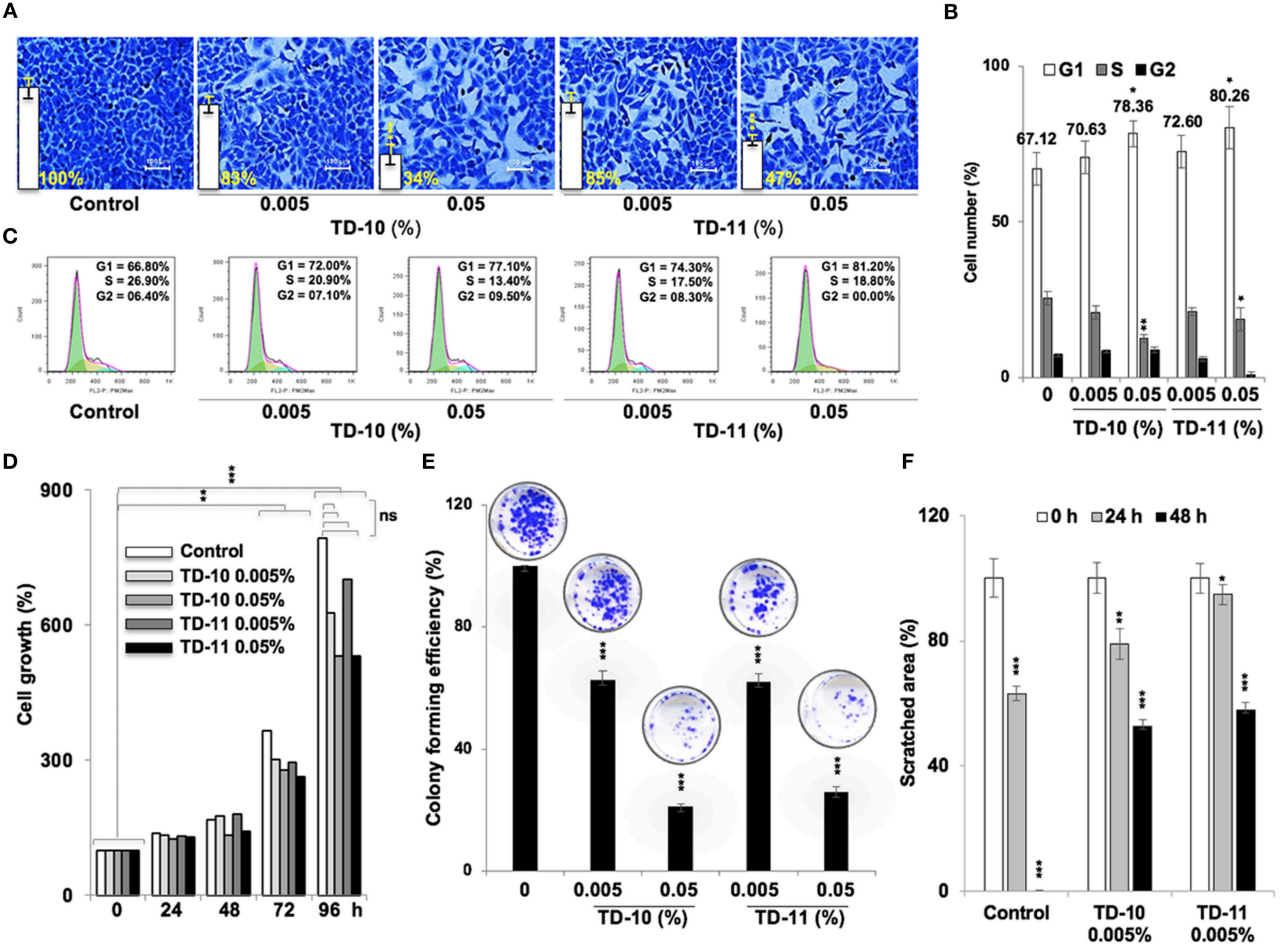
Effect of low doses of TD-10 and TD-11 on cell viability of A549 cells. **(A)** Crystal violet-stained photographs of cells treated with 0.005 and 0.05% of TD-10 and TD-11 are shown. (**B)** Cell cycle profile of control and treated cells showing mild growth arrest at G1 phase in 0.005 % TD-10 and TD-11 treated (48 h) cells, and lack of apoptosis. (**C)** Consolidated data from three independent experiments showing dose dependent accumulation of cells in G_1_ phase of the cell cycle in TD-10 and TD-11 treated cells. (**D)** Growth curve of cells treated (48 h) with low doses of TD-10 and TD-11 showing lack of toxicity for 0.005% TD-10 and TD-11 during 48 h treatment. **(E)** Colony forming efficiency of control, TD-10 and TD-11 treated (10 days) cells showing dose dependent decrease. (**F)** Migration capacity of cells treated with non-toxic conditions of TD-10 and TD-11 treatment. Both TD-10 and TD-11 caused inhibition in cell migration. Images of Wound-Scratch assay are shown in Supplementary Figure S2a. Statistical analysis from three independent experiments is depicted as *p < 0.05, **p < 0.01, ***p < 0.001, and ns = not significant.

In view of anti-migration, anti-invasion and anti-angiogenesis activities of TEG (Figure 1), we next investigated such activities in response to TD-10 and TD-11 treatment. As shown in Figure 3A, TD-10 and TD-11 caused stronger inhibition of cell invasion as compared to TEG. More importantly, TD-10 (0.5, 1.0, and 2.5%) and TD-11 (1.0 and 2.5%) induced markedly greater inhibition of cell invasion than Avastin. Tube formation assay also revealed stronger effect of TD-10 and TD-11 as compared to TEG (Figure 3B) up to the dose of 0.1%. Higher doses such as 0.25 and 0.5% with strong cytotoxicity in cell viability assays (Figure 2B) showed no tube formation as well. In order to differentiate the effect of TD-10 and TD-11 on cell proliferation and cell migration capacities, we determined their non-toxic doses by extensive cell viability and cell cycle analyses (Figure 4). While both the derivatives caused significant growth arrest at 0.05%, the effect of 0.005% was milder and mostly non-significant in short-term (48 h) assays (Figures 4A-D). Long-term viability (4 days) assays showed very mild, but notable, effect of 0.005% TD-10 and TD-11 on growth (Figures 4D). Statistical analyses of cell growth from three independent experiments further revealed that the effect was statistically insignificant. Colony formation (8–10 days) assay, on the other hand, exhibited statistically significant reduction in colony number in cultures treated with both 0.005 and 0.05% TD-10 and TD-11(Figure 4E). In several independent experiments, 0.05 and 0.005% dose of both TD-10 and TD-11 caused ~70 and 40% reduction in clonogenicity. Based on these data, we considered 0.005% (TD-10 = 0.17 mM, and TD-11 = 0.15 mM) as non-toxic and suitable for Wound-Scratch assays that were monitored at 24 and 48 h. As shown in Figure 4F, Supplementary Figure S3a, the TD-10 and TD-11 (0.005%) were seen to cause significant inhibition in cell migration.

**Figure 5:**
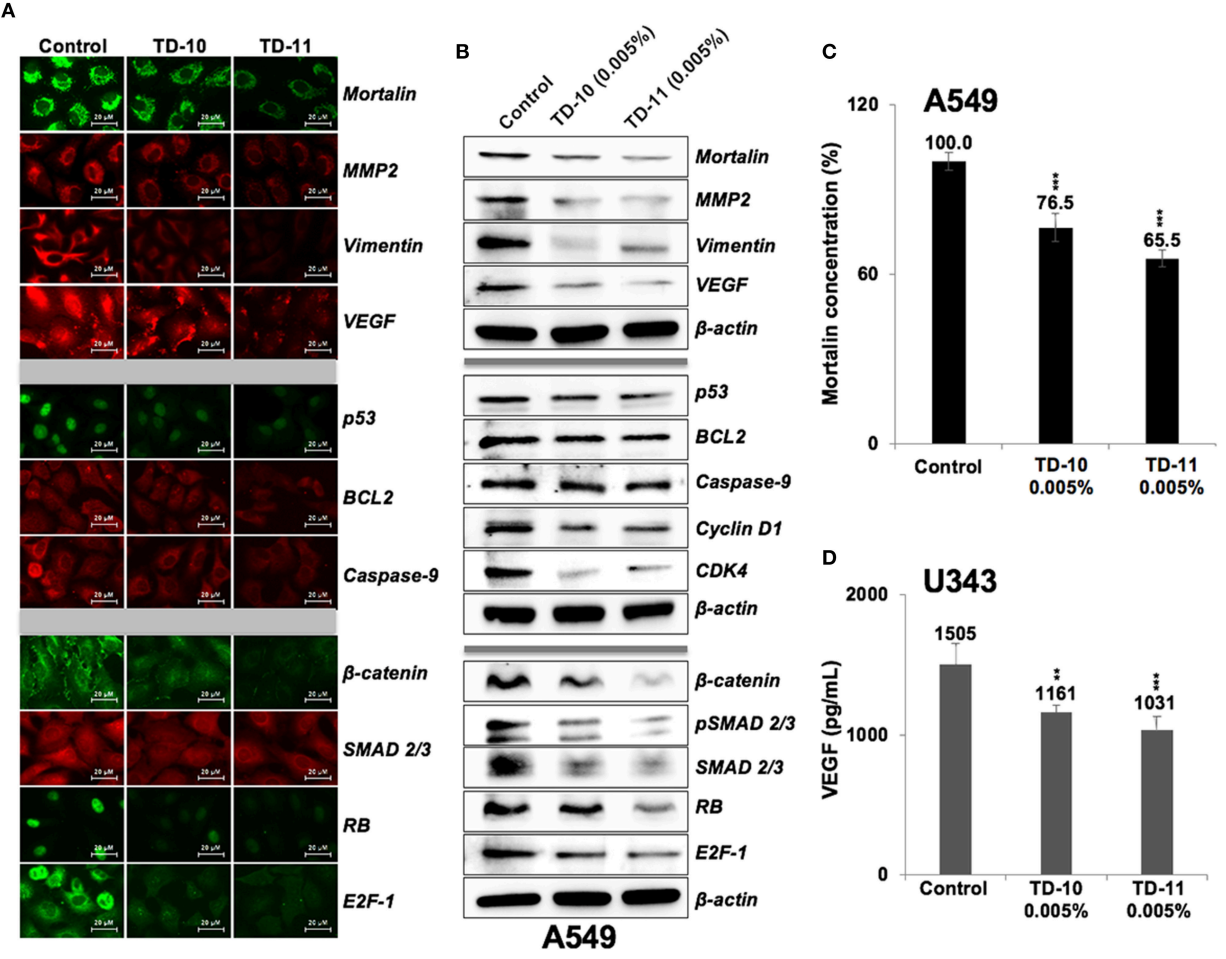
Effect of TD-10 and TD-11 (0.005%; 48 h) on proteins involved in cell migration, apoptosis and proliferation. (**A,B)** Immunostaining and immunoblotting of A549 cells treated with TEG derivatives. Downregulation of proteins involved in cell migration and EMT (mortalin, MMP2, vimentin, VEGF, β-catenin, SMAD2/3) was recorded. Downregulation of phosphorylated RB and E2F-1, Cyclin D1, and CDK4 signified moderate inhibition of cell proliferation and migration. Changes in p53, BCL2, Caspase 9 expression were mild and signified lack of apoptosis. (**C,D)** Mortalin and VEGF ELISA revealed downregulation in both TD-10 and TD-11 treated cells. Statistical analysis is depicted as *p < 0.05, **p < 0.01, ***p < 0.001.

We next performed expression analyses of several proteins involved in cell migration and invasion. As shown in Figure 5A, significant downregulation of mortalin, MMP2, vimentin, and VEGF (major regulators of cell invasion capacity and angiogenesis) was observed in cells treated with 0.005% TD-10 and TD-11 (Figures 5A,B, Supplementary Figure S4). However, although Mortalin ELISA also confirmed decrease in mortalin in response to TD-10 and TD-11 treatment (Figure 5C), it was not associated with increase in p53 as expected (15, 17, 29). Instead, we observed decrease in p53 (mutant) in A549 cells suggesting that TD-10 and TD-11 treatment may affect cell proliferation and migration by p53-independent pathways. We also examined the key regulators of apoptosis and found only a slight decrease in Bcl-2, and Procaspase 9. These data were consistent with the microscopic observations endorsing that TD-10 and TD-11 (0.005%) did not induce apoptosis. On the other hand, regulators of cell migration including β-catenin, SMAD2/3 and its activated phosphorylated form (pSMAD2/3), pRB, E2F1, Cyclin D1, and CDK4 showed downregulation in treated cells (Figure 5B, Supplementary Figure S4) supporting the decrease in their migration and invasion capacities. We also examined the expression of VEGF specifically by ELISA in control and treated U343 cells (29) and found significant reduction in the latter suggesting the effect was not cell line specific (Figure 5D).

**Figure 6:**
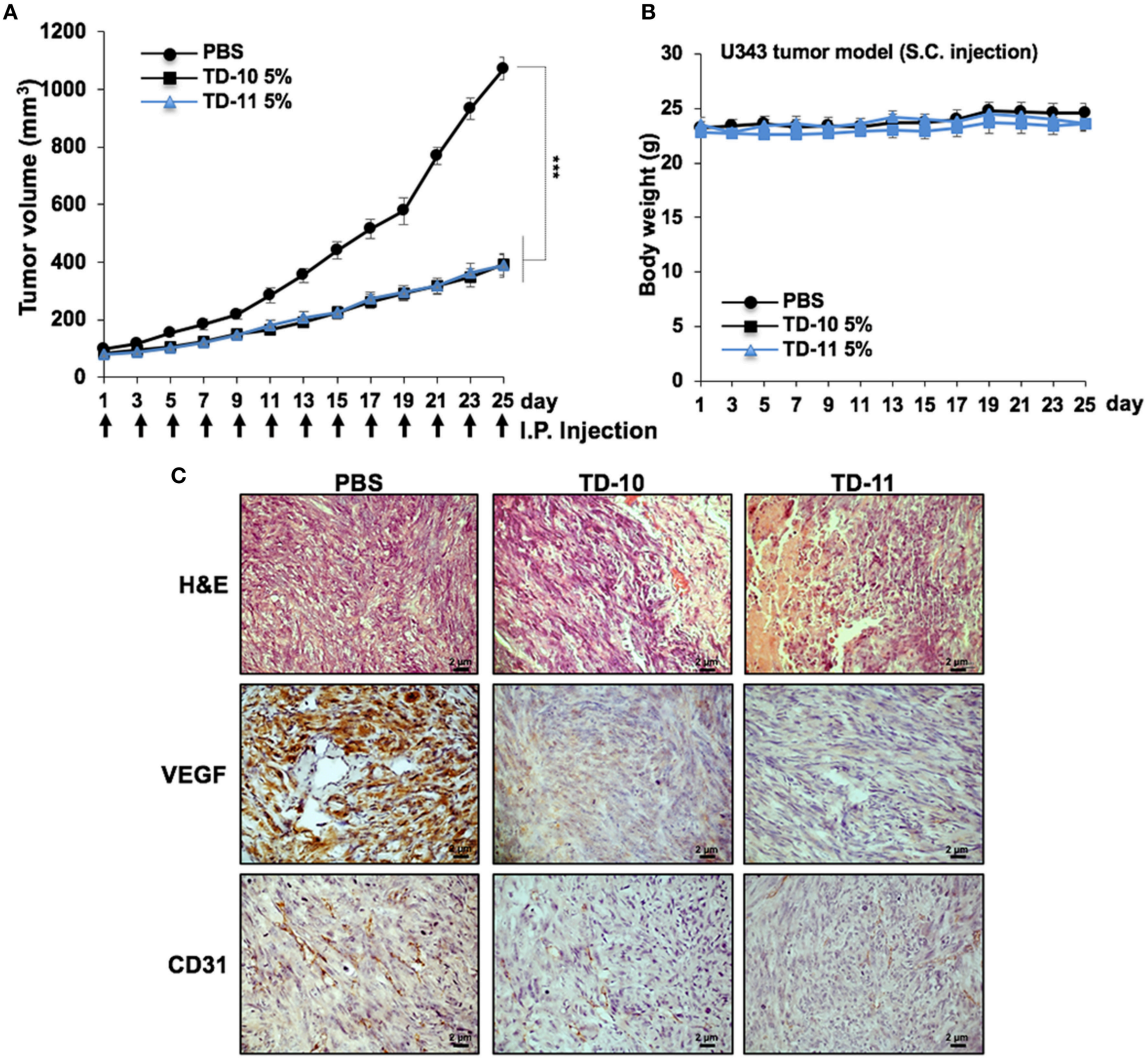
*In vivo* tumor progression assays using U343 cells in subcutaneous xenograft model. (**A)** TD-10 and TD-11 mice showed significant delay in tumor growth. (**B)** Body weight of control and treated mice did not show any difference. (**C)** Immunohistochemistry showing decrease in VEGF and CD31 expression in treated mice as compared to control.

We next performed *in vivo* tumor progression assays in nude mice subcutaneous xenografts. Both TD-10 and TD-11 (5%) did not show any hemolytic activity (Supplementary Figure S3b). As shown in Figure 6A, TD-10 and TD-11 caused strong inhibition of tumor progression. There was no significant change in body weight in mice treated with either TD-10 or TD-11 (Figure 6B). Furthermore, tumor sections from TD-10 and TD-11 treated mice showed decrease in the level of VEGF and CD31 (markers for angiogenesis) expressions (Figure 6C), suggesting strong inhibition of angiogenesis. Similar results were obtained in nude mice using A549 cells in subcutaneous xenografts as well as tail vein lung metastasis models along with oral feeding of TD-10 and TD-11 (Supplementary Figures S5a). As compared to the control group, the treated groups showed significant reduction in size of subcutaneous as well lung metastasized tumors (Supplementary Figure S5b).

## Discussion

We earlier identified TEG as an active anticancer component in the water extract of leaves of Ashwagandha (WEX). It was seen to activate tumor suppressor proteins p53 and pRB resulting in growth arrest of cancer cells (17). In order to recruit WEX and TEG as anti-metastasis reagents, we investigated their potential in *in vitro* assays that determine the cell migration, invasion and angiogenic potentials. VEGF, as an established key regulator of metastasis and angiogenesis (30) was used as an enhancer of these phenotypes. We found that it caused mild increase in migration, invasion and tube formation capacity of cells, and in contrary both WEX and TEG strongly inhibited these phenotypes (Figures 1A,C). Cell invasion ability showed considerable increase in VEGF-treated cells but inhibited in response to WEX and TEG treatments (Figure 1B). Of note, the effect was comparable with Avastin and FP3, well-established inhibitors of angiogenesis and metastasis. We next examined the TEG derivatives in search of more suitable anticancer reagents.

*In vitro* cell viability assays showed cytotoxicity of TD-5, TD-8, TD-10, and TD-11 in different cancer cell lines (Figure 2A). Based on these data and dose dependent-cytotoxicity assays, we selected TD-10 and TD-11 for molecular analyses. Both these were seen to inhibit cell migration and angiogenesis better than TEG and comparable to the positive control (Avastin) in aggressive non-small lung cancer A549 cells. Cell cycle and growth analyses revealed that both TD-10 and TD-11 at 0.05% dose caused growth arrest in G_1_ phase, inhibited the proliferation and clonogenicity (Figures 4A,D,E). On the other hand, treatment for 48 h with both TD-10 and TD-11 at 0.005% dose showed milder effect on cell cycle progression and no change in cell growth (Figures 4B,C). Colony forming assays that required continued treatment for ~10 days showed ~40 % inhibition of colonogenicity for both TD-10 and TD-11 (Figures 4E). Based on these data, we considered treatment with TD-10 and TD-11 at 0.005% concentration for 24–48 h as non-toxic, and investigated their effect on cell migration by Wound-Scratch assays. Both of these were seen to delay the migration of cells to the scratch (Figure 4F, Supplementary Figure S3a) and was supported by downregulation of mortalin (stress chaperone that has been shown to play role in carcinogenesis, EMT, and cancer cell stemness) (30), MMP2 (gelatinase-2 that degrade extracellular matrix viz., type 4 collagen and regulate the cell invasion ability) (31), Vimentin (a type 3 intermediate filament protein mainly expressed in mesenchymal origin cells, contributing to the maintenance of cell cytoskeleton, anchorage, integrity, migration, and angiogenesis) (32) and VEGF (an angiogenic factor and key regulator of cancer cell invasion and angiogenic properties) (30) (Figures 5). Furthermore, consistent with the phenotype analyses, we found that the apoptosis-regulatory proteins were not altered significantly. On the other hand, distinct decrease in the expression of β-catenin, SMAD2/3, RB and E2F1 was in agreement with diminished migration, invasion and angiogenic properties (33), as sought earlier (Figures 5A,B, Supplementary Figure S5). Wnt/β-catenin pathway is one of the key regulators of cell survival, proliferation and EMT, a cadherin-driven process by which the immobile epithelial cells transform their morphology into fibroblastic and motile mesenchymal type (34). It is co-activated by TGF-β activated SMAD2 via a transactivation crosstalk with TCF4 (33). Upregulated β-catenin triggers transcriptional activation of SNAIL2-mediated EMT and proliferation signaling (31, 35). TD-10 and TD-11 caused decrease in β-catenin implying inhibition of the canonical Wnt/β-catenin pathway yielding attenuation of the EMT as well as cell proliferation signaling. Decrease in SMAD 2/3 and its activated phosphorylated form endorsed the inhibition of EMT. Consistent with the Western blot data, immunofluorescence results also revealed decrease in staining of both β-catenin and SMAD 2/3 signifying suppressed EMT signaling in treated cells. However, no distinct change in the subcellular distribution of SMAD 2/3 was observed in treated cells. On the other hand, Vimentin, MMP2, mortalin, and VEGF showed remarkable decrease, agreeing with the inhibition of cell migration, invasion and angiogenic phenotypes. *In vivo* results in Balb/c nude mice validated that TD-10 and TD-11 possess potential tumor growth suppressor and anti-migratory activities (Figure 6, Supplementary Figure S4). Consistent with the *in vitro* anti-angiogenic activities, tumors from the TD-10 and TD-11 treated mice showed decrease in VEGF and CD31 expression and validated their anti-angiogenic potential *in vivo*.

Triethylene amine and some of its derivatives have been in use in dental and chemical solvent industries. TD-1 was recently shown to upregulate virulence associated genes and proteins in *S. mutans* sp. (36), hence understanding the mechanisms of action and development of these derivatives could prove to be extremely beneficial. While biological effects of TD-11, a diester formed by the condensation of methacrylic acids and ethylene glycol (33), used mainly as a synthetic co-polymer have not been investigated, TD-10 is traditionally used in post-operative care in dentistry as a resin layer to temporarily cover the dental composite materials upon polymerization (37). Cytotoxicity of TD-10 has also been reported in some other studies. Batarseh et al. (35) demonstrated induction of apoptotic signaling in human pulp fibroblasts by TD-10. By analyzing human apoptotic array, they found that TD-10 treated cells showed sustained upregulation of BH3 family proteins, caspase 3/8, and cytochrome c release. Krifka et al. (37) reported induction of ROS in human transformed pulp derived cells, cervical adenocarcinoma cells and mouse macrophages in response to the treatment with TD-10. Similarly, TD-10 induced ROS production and activation of apoptotic signaling via Cytochrome c release and upregulation of BCL-XL and caspase 9 was reported in human embryonic palatal mesenchymal and dental pulp cells (38) and in normal skin fibroblasts (39). Human gingival fibroblasts (40) showed cytotoxicity due to TD-10, mediated by upregulation of p21 and G_2_/M cell cycle arrest in a p53-independent manner (41). Similar effects were observed in normal human skin fibroblasts and primary dental pulp cells, and were ascribed to upregulation of p21 and downregulation of CDC2, Cyclin B1, and CDC25C (42). It was also shown to induce upregulation of COX-2, PGE_2_, and PGE_2α_ expression suggesting its role as a pro-inflammatory molecule (43). TD-10-mediated cytotoxicity caused by excessive production of ROS was blocked by N-acetylcysteine (NAC) pre-treatment, in which NAC was shown to make TEGDMA-NAC adducts in extracellular and intracellular compartments (44). TD-10 induced large deletions in the *hprt gene* (45) that plays a key role in purine salvage pathway and generation of purine nucleotides, and its mutations may result in inflammatory disorders such as Lesch-Nyhan syndrome and gout (46). TD-10 at 1 mM dose caused 76% complete and 20% partial deletions in Chinese hamster cell lines. Our data suggested dose dependent anti-migration, anti-invasion and anti-proliferation potency of TD-10 and TD-11 that may be explored for their recruitment as treatment of metastatic cancers.

TD-10 and TD-11 are methacryloyl derivatives of TEG (TD-1) that may account for their improved cytotoxicity in the biological system (47). While both are the hydration products of ethylene oxide, produced when ethylene is oxidized at high temperature in the presence of silver oxide as a catalyst, structural difference between TD-10 and TD-11 is only the length of chain composed of ethylene glycol groups (Supplementary Figure 1). Lighter chemical chain of TD-10 may furnish increased permeability and dissolution rate that may translate into higher cytotoxicity than TD-11 (48). On the other hand, higher stability of TD-11 may account for its stable and longer effects. These predictions and our data call for further studies to explore the biochemical and molecular mechanisms of anti-proliferative, anti-migration and anti-angiogenic activities of TD-10 and TD-11 to develop them as anti-metastasis drugs.

## Author contributions

EO, C-OY, SK, and RW were responsible for the study concept and design. C-OY, SK, and RW obtained funding. EO, SG, YL, and SA acquired data. EO, SG, SA, C-OY, and RW analyzed and interpreted data. EO, SG, SA, C-OY, SK, and RW drafted the manuscript, and all authors revised it for important intellectual content.

### Conflict of interest statement

The authors declare that the research was conducted in the absence of any commercial or financial relationships that could be construed as a potential conflict of interest.

## Supplementary material

Supplementary Figure S1 Structures of TEG and its 10 derivatives are shown.

Supplementary Figure S2 Short-term cytotoxicity evaluation of TD-10 and TD-11. Cell viability of control, TD-10 and TD-11 treated (48 h) cells as performed with **(a)** QCV (**b**) AlamarBlue™ and **(c)** ATP-glo™ assays. All the three assays demonstrated a consistent and comparable dose-dependent cytotoxicity pattern in A549 cells treated (48 h) with TD-10 and TD-11. Statistical analysis is depicted as ^*^*p* < 0.05, ^**^*p* < 0.01, ^***^*p* < 0.001.

Supplementary Figure S3 Anti-migratory and anti-hemolytic profile of TD-10 and TD-11. (**a**) Images showing time dependent migration of A549 cells cultures in control, TD-10 and TD-11 supplemented medium in Wound-Scratch assays are shown. Wound edges are marked with dotted lines. (**b)** Hemolytic activity of TEG (TD-1), TD-10 and TD-11 is shown. Statistical analysis is depicted as ^*^*p* < 0.05, ^**^*p* < 0.01, ^***^*p* < 0.001.

Supplementary Figure S4 Quantitation of the protein expression in control, TD-10, TD-11 treated cells. Quantitation of proteins as obtained from immunocytochemistry (**a**) and Western blot (**b**) analyses of control, TD-10 and TD-11 at their non-toxic doses in correspondence to Figures 5A,B. Twenty-thirty cells/field from three fields/experiment from three independent experiments for immunostaining and band imaged from three independent experiments for immunoblotting were analyzed for quantitation. Statistical analysis is depicted as ^*^*p* < 0.05, ^**^*p* < 0.01, ^***^*p* < 0.001.

Supplementary Figure S5 *In vivo* tumor suppressor assays using A549 cells in subcutaneous xenograft and tail vein metastasis model. (**a)** TD-10 and TD-11 mice showed suppression of tumor growth. (**b)** Morphological inspection of dissected lungs showed reduced number of metastasized cells and size of developed tumor in mice treated with TD-10 and TD-11. Statistical analysis is depicted as ^*^*p* < 0.05, ^**^*p* < 0.01, ^***^*p* < 0.001.
