# Supplementary figures and images for: Identification and Functional Characterization of Anti-metastasis and Anti-angiogenic Activities of Triethylene Glycol Derivatives
Source: Front Oncol. 2018 Nov 28;8:552. doi: 10.3389/fonc.2018.00552 (PMC6279921; doi:10.3389/fonc.2018.00552)

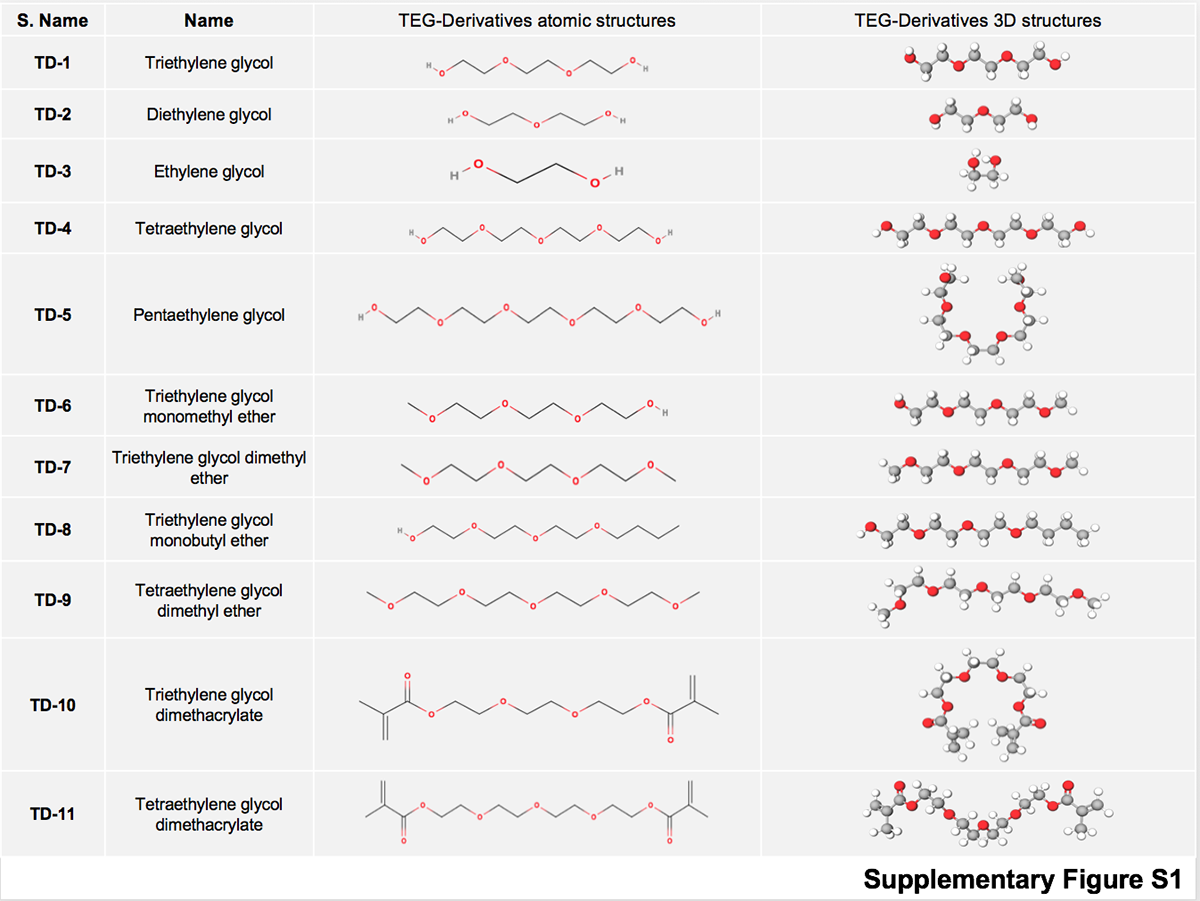

Supplement: Supplementary Figure S1 — Structures of TEG and its 10 derivatives are shown. [file Image_1.TIF]

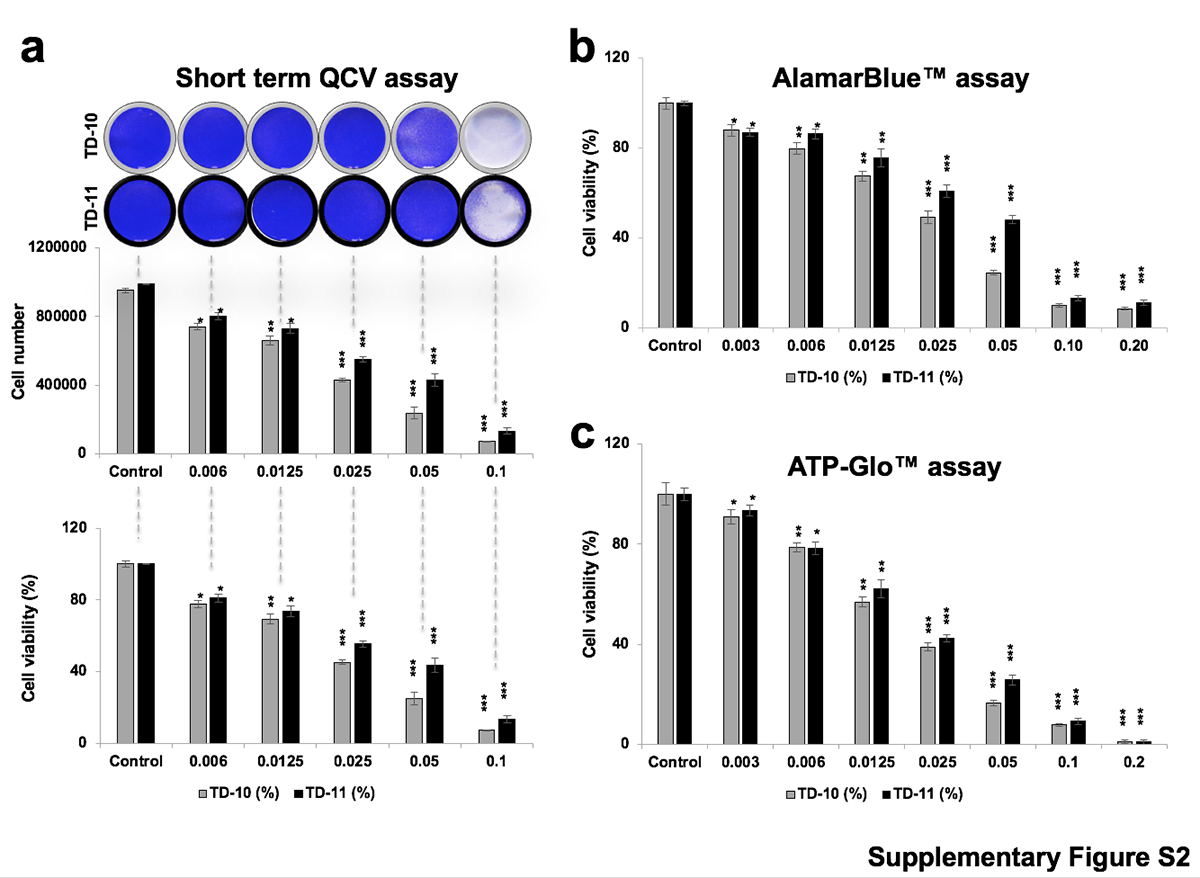

Supplement: Supplementary Figure S2 — Short-term cytotoxicity evaluation of TD-10 and TD-11. Cell viability of control, TD-10 and TD-11 treated (48 h) cells as performed with (a) QCV (b) AlamarBlue™ and (c) ATP-glo™ assays. All the three assays demonstrated a consistent and comparable dose-dependent cytotoxicity pattern in A549 cells treated (48 h) with TD-10 and TD-11. Statistical analysis is depicted as *p < 0.05, **p < 0.01, ***p < 0.001. [file Image_2.TIF]

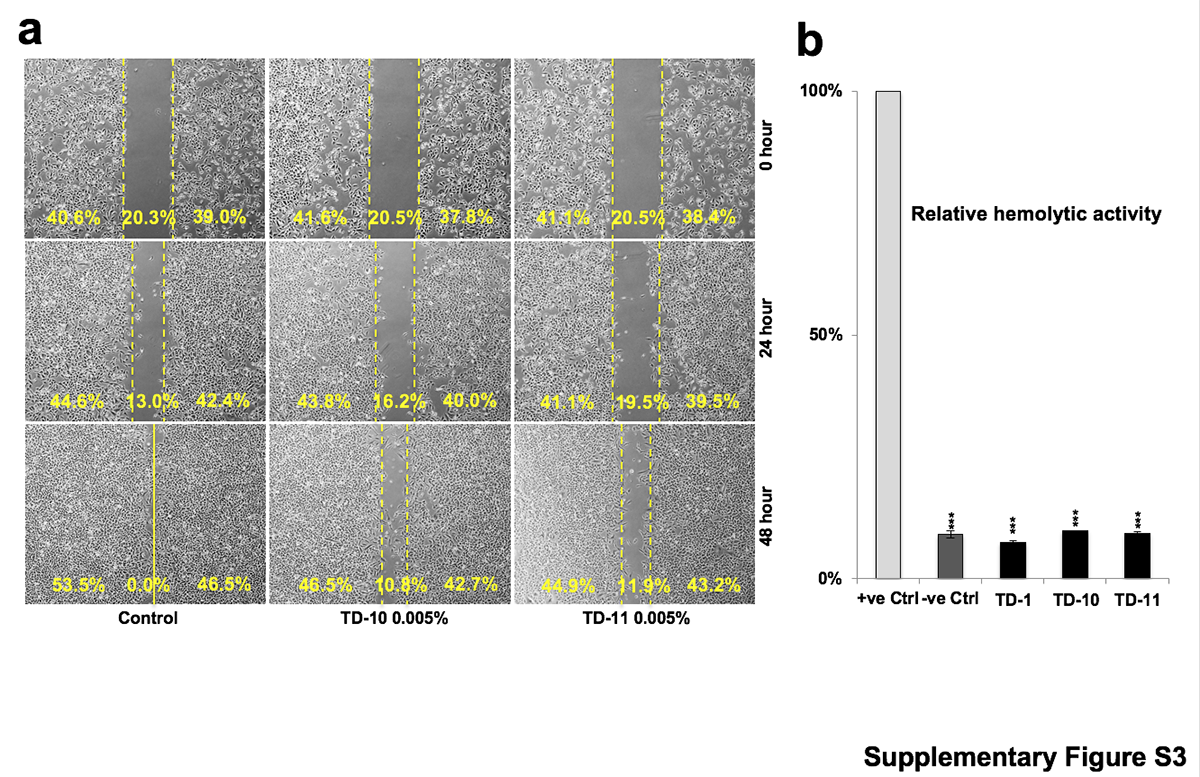

Supplement: Supplementary Figure S3 — Anti-migratory and anti-hemolytic profile of TD-10 and TD-11. (a) Images showing time dependent migration of A549 cells cultures in control, TD-10 and TD-11 supplemented medium in Wound-Scratch assays are shown. Wound edges are marked with dotted lines. (b) Hemolytic activity of TEG (TD-1), TD-10 and TD-11 is shown. Statistical analysis is depicted as *p < 0.05, **p < 0.01, ***p < 0.001. [file Image_3.TIF]

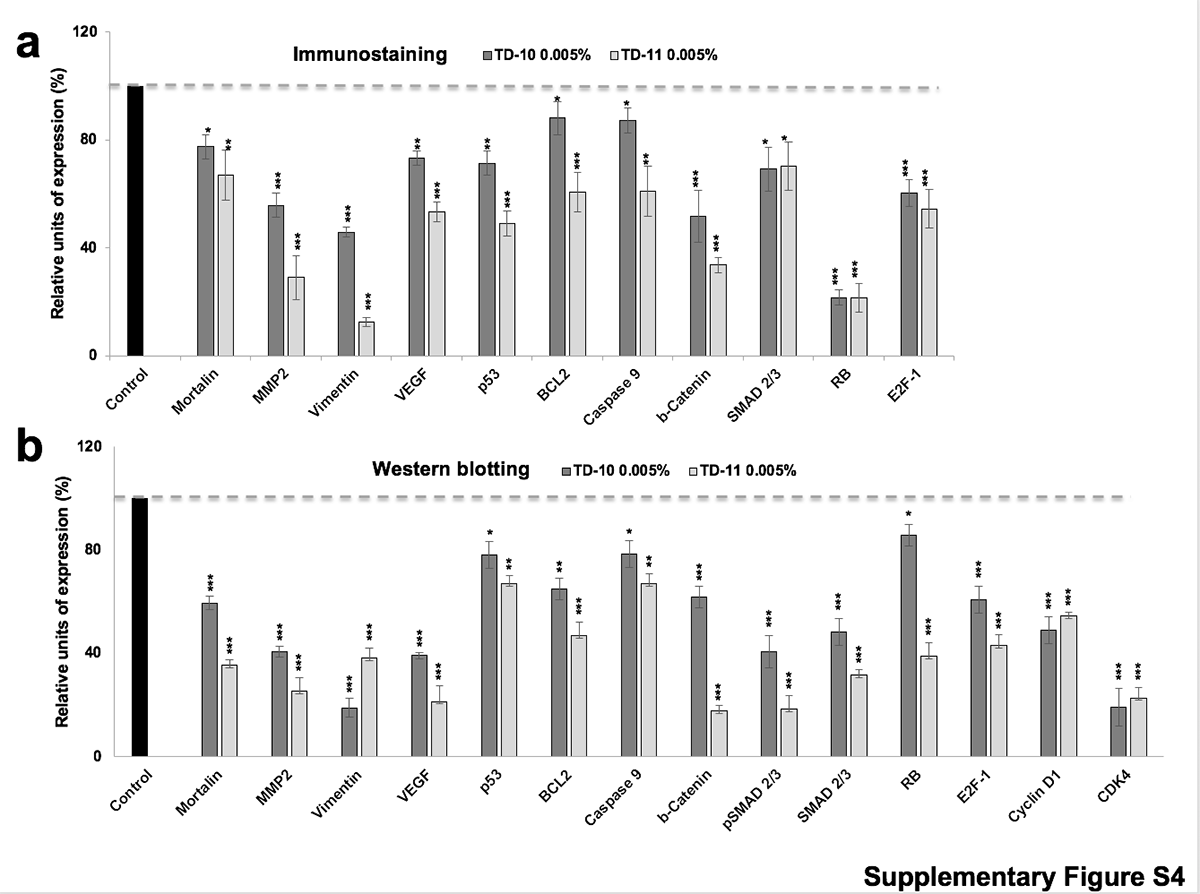

Supplement: Supplementary Figure S4 — Quantitation of the protein expression in control, TD-10, TD-11 treated cells. Quantitation of proteins as obtained from immunocytochemistry (a) and Western blot (b) analyses of control, TD-10 and TD-11 at their non-toxic doses in correspondence to Figures 5A,B. Twenty-thirty cells/field from three fields/experiment from three independent experiments for immunostaining and band imaged from three independent experiments for immunoblotting were analyzed for quantitation. Statistical analysis is depicted as *p < 0.05, **p < 0.01, ***p < 0.001. [file Image_4.TIF]

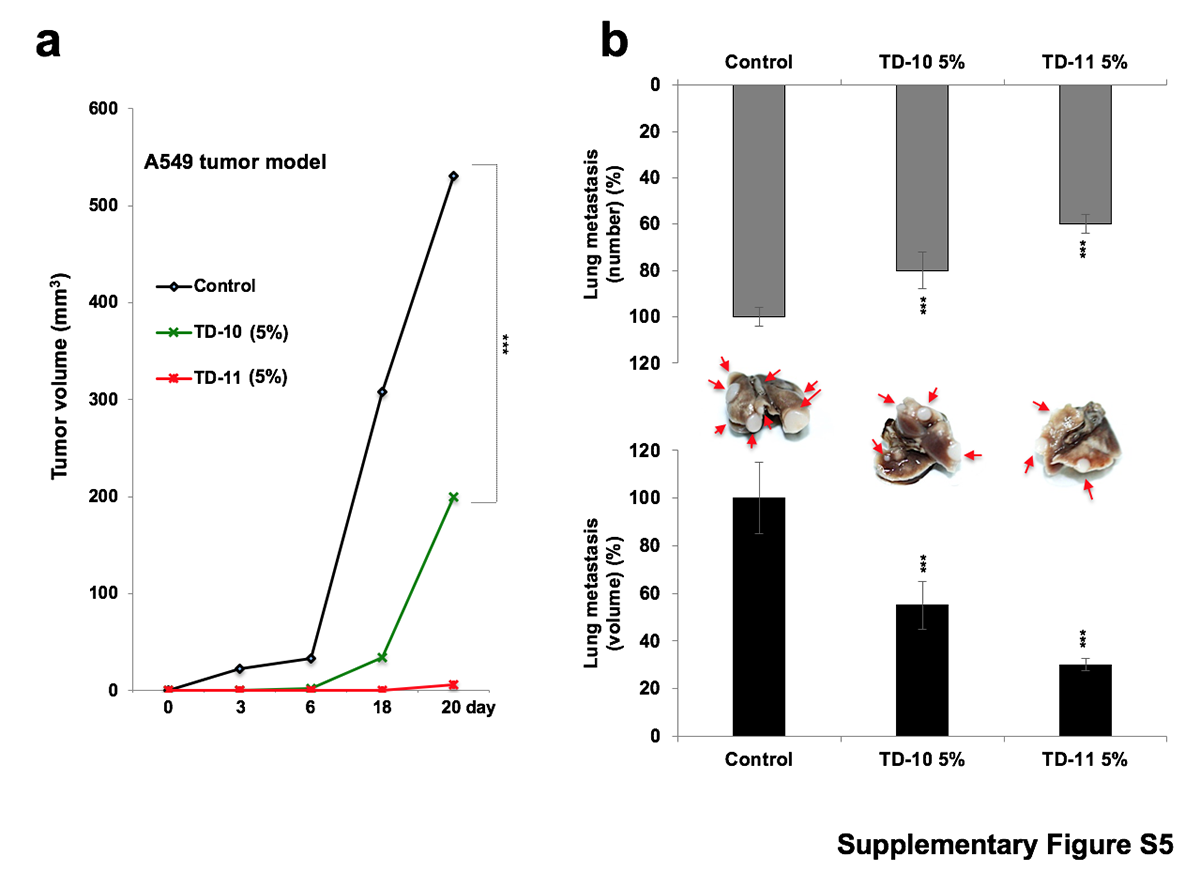

Supplement: Supplementary Figure S5 — In vivo tumor suppressor assays using A549 cells in subcutaneous xenograft and tail vein metastasis model. (a) TD-10 and TD-11 mice showed suppression of tumor growth. (b) Morphological inspection of dissected lungs showed reduced number of metastasized cells and size of developed tumor in mice treated with TD-10 and TD-11. Statistical analysis is depicted as *p < 0.05, **p < 0.01, ***p < 0.001. [file Image_5.TIF]
